# Immune Checkpoint Inhibition in Metastatic Colorectal Cancer Harboring Microsatellite Instability or Mismatch Repair Deficiency

**DOI:** 10.3390/cancers13051149

**Published:** 2021-03-08

**Authors:** Romain Cohen, Raphaël Colle, Thomas Pudlarz, Maximilien Heran, Alex Duval, Magali Svrcek, Thierry André

**Affiliations:** 1 Department of Medical Oncology, Assistance Publique des Hôpitaux de Paris (AP-HP), Saint-Antoine Hospital, Sorbonne Université, F-75012 Paris, France; raphael.colle@aphp.fr (R.C.); thomas.pudlarz@aphp.fr (T.P.); maximilien.heran@aphp.fr (M.H.); thierry.andre@aphp.fr (T.A.); 2 Centre de Recherche Saint Antoine, Équipe Instabilité des Microsatellites et Cancer, Équipe Labellisée par la Ligue Nationale Contre le Cancer et SIRIC CURAMUS, INSERM UMRS 938, Sorbonne Université, F-75012 Paris, France; alex.duval@inserm.fr (A.D.); magali.svrcek@aphp.fr (M.S.); 3 Department of Pathology, Assistance Publique des Hôpitaux de Paris (AP-HP), Hôpital Saint-Antoine, Sorbonne Université, F-75012 Paris, France

**Keywords:** microsatellite instability, colorectal cancer, deficient mismatch repair, Lynch syndrome, immunotherapy

## Abstract

**Simple Summary:**

Microsatellite instability (MSI) is a molecular indicator of defective DNA mismatch repair (dMMR). MSI/dMMR status is observed in approximately 5% of metastatic colorectal cancers (mCRC) but 10–18% of localized colorectal cancers. MSI/dMMR status is a major predictive biomarker for the efficacy of immune checkpoint inhibitors (ICIs). This review presents the current and future challenges of ICIs for patients with MSI/dMMR colorectal cancer.

**Abstract:**

Microsatellite instability (MSI) is a tumor phenotype related to a deficient DNA mismatch repair system (dMMR). This phenotype, observed in 5% of metastatic mCRC but 10–18% of localized CRC, is associated with high tumor mutational burden with highly immunogenic neoantigens. It has emerged as a major predictive biomarker for the efficacy of ICIs. In this review, we will present a comprehensive overview of the literature concerning the efficacy of ICIs in MSI/dMMR mCRC, with a focus on new developments in first-line metastatic setting. Then, we will present current and future challenges of immuno-oncology for patients with MSI/dMMR metastatic CRC.

## 1. Introduction

The discovery of immune checkpoints and immune checkpoint inhibitors (ICIs) revolutionized the history of medical oncology and was rewarded by the 2018 Nobel Prize in Physiology or Medicine. In the context of cancer immunotherapy has emerged a major predictive biomarker: microsatellite instability (MSI).

The MSI tumor phenotype is caused by a deficiency of the MMR system, resulting from a MMR gene germline mutation (*MLH1*, *MSH2*, *MSH6*, *PMS2*; i.e., Lynch syndrome) or from an epigenetic inactivation of *MLH1* or double somatic mutations in the MMR genes (i.e., sporadic cancers) [1,2]. Sporadic MSI/dMMR colorectal cancers (CRC) are frequently associated with the *BRAF*^V600E^ mutation, through its association with the CpG island methylator phenotype (CIMP) [3].

MSI/dMMR tumors represent approximately 10–18% of localized CRC and 3–5% of metastatic CRC (mCRC) [3,4]. The MSI/dMMR status is associated with favorable outcomes for stage II cancers. While the positive prognostic impact of MSI/dMMR is observed for stage III N1 tumors, patients with stage III N2 CRC experience similar outcomes than patients with MSS/pMMR (microsatellite stable, proficient mismatch repair) tumors. In metastatic setting, the prognostic impact of MSI/dMMR remains unclear [3,5,6].

**Figure 1:**
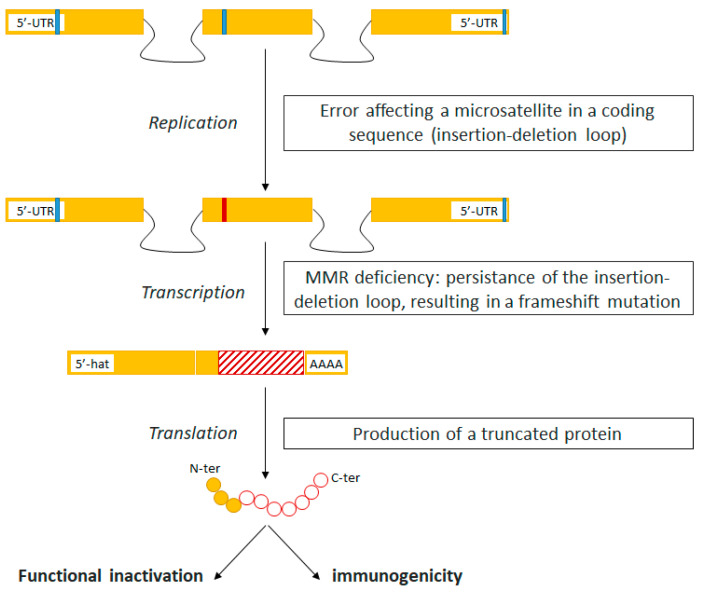
The immunogenicity of tumors with mismatch repair deficiency. Yellow bars: exons; Black lines: introns; Blue dash: non-mutated microsatellite; Red dash: mutated microsatellite.

The MSI-driven oncogenic pathway leads to a high tumor mutational burden, with highly immunogenic neoantigens arising from frameshift mutations. Consequently, MSI tumors are highly infiltrated by cytotoxic T lymphocytes (Figure 1). To escape this hostile microenvironment, MSI tumors exhibit high levels of immune checkpoints, paving the way for therapeutic strategies with ICI [7,8,9]. The proof-of-concept phase II KEYNOTE-016 study was designed to assess the efficacy of pembrolizumab (anti-PD1) in three cohorts of chemoresistant patients: MSI/dMMR mCRC, MSI/dMMR non-CRC, and MSS/pMMR CRC. Preliminary results published in 2015 showed objective response rates (ORR) of 40%, 71%, and 0%, respectively, in the three pembrolizumab-treated cohorts (*n* = 11; *n* = 9; *n* = 21). This was the first step to recognize MSI/dMMR as a major predictive tissue-agnostic biomarker for the efficacy of ICIs [10].

In this review, we will summarize data concerning the clinical activity of ICI in patients with chemoresistant and chemotherapy-naïve MSI/dMMR mCRC. Then, we will focus on the next challenges, notably the resistance of MSI/dMMR cancers to ICI.

## 2. Immune Checkpoint Inhibitors in MSI/dMMR mCRC

### 2.1. In Second Line or Latter

Results from biomarker-guided non-randomized phase II trials are displayed in Table 1. ICI have demonstrated impressive clinical activity in patients with heavily pre-treated MSI/dMMR mCRC. Objective response rates (ORR) range from 28% to 69%, and 2-year progression-free survival (PFS) rates from 37% to 60%. Based on these positive results, pembrolizumab and nivolumab alone or in combination with ipilimumab are approved for the treatment of patients with chemoresistant MSI/dMMR mCRC in the USA.

In the updated analysis of the KEYNOTE-016 study, with 78 evaluable patients, pembrolizumab provided an ORR of 52% and 54% in those with MSI/dMMR CRC and MSI/dMMR non-CRC, respectively [22]. In the KEYNOTE-164 phase II study evaluating pembrolizumab in MSI/dMMR mCRC patients, 44% and 30% of patients from cohort A (≥2 prior lines of standard therapy) and cohort B (≥1 prior line of standard therapy) had received at least 3 prior lines of chemotherapy, respectively. With a median follow-up of 31 months and 24 months, the objective response rate was 33% in both cohorts. Median duration of response was not reached. Respectively, 16% of 13% of patients from cohorts A and B had treatment-related grade 3–4 adverse events [13].

Results of CHECKMATE-142 phase II study confirmed ICI as a breakthrough therapeutic strategy for MSI/dMMR mCRC [15,17]. The trial evaluated nivolumab with and without ipilimumab in two distinct cohorts of patients previously pretreated for MSI/dMMR mCRC. Most (54%) of the 74 patients treated by nivolumab monotherapy (3 mg/kg) were heavily pretreated (≥3 prior lines of treatment). ORR and disease control rate (DCR) with nivolumab alone were 34% and 62%, respectively. With a median follow-up of 21 months, medians PFS and overall survival (OS) were not reached at the time of analysis [23]. Clinically meaningful improvements in functioning, symptoms, and quality of life (QoL) were reported. Grade 3–4 treatment-related adverse events (TRAE) occurred in 20% of patients [15]. Of the 119 MSI/dMMR mCRC patients treated with nivolumab plus ipilimumab in the CHECKMATE 142 study, 76% had at least two prior lines of therapy [16]. Patients received nivolumab 3 mg/kg and ipilimumab 1 mg/kg every 3 weeks for 4 doses and then nivolumab 3 mg/kg IV every 2 weeks until disease progression, discontinuation due to toxicity, death, withdrawal of consent, or study end. Eastern Cooperative Oncology Group performance status (ECOG PS) was 0 or 1. With a median follow-up of 25.4 months, nivolumab plus ipilimumab was associated with an ORR of 58% and a 1-year PFS of 71%. Grade 3–4 TRAEs occurred in 32% of these patients [24]. In the phase II GERCOR NIPICOL study, 57 MSI/dMMR mCRC patients were treated with nivolumab and ipilimumab according to the CheckMate 142 schedule, but with a maximum of one year of treatment. Like in the latter study, the 6-month and 1-year PFS rates were 75% and 71%. A total of 56% of the patients experienced grade 3–4 AEs [19]. Dostarlimab, another monoclonal antibody targeting PD-1, showed strong and durable antitumor activity in patients with dMMR gastrointestinal tumors in the phase 1/2b GARNET study. Patients received dostarlimab at 500 mg every 3 weeks for four cycles, and 1000 mg of dostarlimab every 6 weeks thereafter for up to 2 years. In this trial, 69 (65%) patients had mCRC and received previously oxaliplatin, fluoropyrimidines and irinotecan. Treatment-related adverse events were reported in 69% of patients, of which 8% were grade ≥3; treatment-related serious adverse events were observed in 6% of patients, and toxicities leading to discontinuation of treatment occurred in 4%. No deaths associated with dostarlimab were reported. The response rate for mCRC was 36.2% (25.0–48.7%). Median duration of response was not reached (range: 1.74–21.88 months) for the whole population (mCRC and others) and the probability of maintaining a response at 12 and 18 months was 91.0% and 80.9%, respectively [21].

These impressive results from non-randomized studies have to be put in perspective with survival outcomes observed in MSI/dMMR mCRC patients treated with conventional chemotherapy. Conventional treatments seem to display less clinical activity among MSI/dMMR compare to MSS/pMMR mCRC patients. In a pooled analysis of the CAIRO, CAIRO2, COIN, and FOCUS trials, median PFS of first-line chemotherapy was 6.2 months in the MSI/dMMR population compared to 7.6 months in the MSS/pMMR group (HT = 1.33, 95% CI 1.12–1.57) [3]. In a large retrospective multicenter French study of 342 MSI/dMMR mCRC patients, median PFS and overall survival (OS) on first-line chemotherapy were 6.0 and 26.3 months. For second-line chemotherapy, median PFS and OS were 4.4 and 21.6 months [25]. The only ongoing randomized trial comparing ICI to chemotherapy in second line or latter for MSI/dMMR mCRC patients is the randomized phase II PRODIGE 54-SAMCO study (NCT03186326) that compares avelumab, an anti-PD-L1 monoclonal antibody, to standard chemotherapy in second-line setting for patients with MSI/dMMR mCRC. There is no planned crossover to avelumab in case of disease progression with conventional chemotherapy.

### 2.2. In First-Line Metastatic Setting: The KEYNOTE-177 Phase III Study

The efficacy of ICIs has also been demonstrated as front-line treatment for patients with chemotherapy-naive MSI/dMMR mCRC. In a third cohort of the CheckMate-142 trial, 45 patients with no prior treatment for MSI/dMMR mCRC were treated with nivolumab plus ipilimumab. After a median follow-up of 29 months, the ORR was 72%, and the 2-year PFS and OS rates were 57% and 79%, respectively [18].

Importantly, the phase III KEYNOTE-177 trial evaluating first-line treatment of pembrolizumab in patients with MSI/dMMR mCRC met one of its dual primary endpoints of PFS [26]. Patients were randomized between pembrolizumab 200 mg Q3W for a maximum of 35 infusions and standard-of-care first-line chemotherapy (an investigator’s choice of mFOLFOX6 or FOLFIRI with or without bevacizumab or cetuximab). 25% of patients had *BRAF*^V600E^-mutated MSI/dMMR mCRC. Based on an interim analysis, pembrolizumab monotherapy demonstrated a statistically significant and clinically meaningful improvement in PFS compared to chemotherapy. Median PFS using RECIST v1.1 criteria was 16.5 months versus 8.2 months, respectively (HR = 0.60, 95% CI 0.45–0.80); 2-year PFS rate was 48.3% versus 33.1%. The superiority of pembrolizumab was consistent among all subgroups, except patients with *RAS*-mutated tumors (HR = 1.19, 95% CI 0.68–2.07), which requires deeper investigations and data for OS. Importantly, immediate disease progression was observed in 29.4% of patients treated with pembrolizumab versus 12.3% with chemotherapy. Misdiagnosis of MSI/dMMR status and pseudoprogression phenomenon (pseudoprogression represents up to 50% of patients with immediate disease progression [27]) might partly explain this observation [19,27,28,29]. Grade 3 or higher treatment-related adverse events were 21.6% versus 65.7%, including death in one patient receiving chemotherapy. Nonetheless, given these results, biomarkers of ICI resistance among MSI/dMMR mCRC seem urgently needed.

It is noteworthy patients randomly assigned to chemotherapy in the KEYNOTE-177 study could cross over to pembrolizumab after disease progression. At the time of data cutoff, the effective crossover rate was 59% in the intention-to-treat population, which might hamper the statistical power of the OS analysis. The PFS2, defined by time from randomization to progression on next line of therapy (second line if received),or death was not reached in the pembrolizumab arm and 23.5 moths in the chemotherapy arm [30]. Pembrolizumab monotherapy led to clinically meaningful improvements in health-related quality of life compared with chemotherapy in patients with previously untreated MSI-H/dMMR mCRC [31].

All in all, pembrolizumab is highly likely to become the new standard of care first-line treatment for patients newly diagnosed with MSI/dMMR mCRC. On June 29, 2020, the Food and Drug Administration approved pembrolizumab for the first-line treatment of patients with unresectable or metastatic microsatellite instability-high (MSI-H) or mismatch repair deficient (dMMR) colorectal cancer and on January 28, 2021, the European Medicine Evaluation Agency (EMEA) approved pembrolizumab for the same indication in Europe.

Data from the CA209-8HW phase III (NCT04008030) study and the COMMIT phase III trial (NCT02997228) are awaited to obtain a clearer a picture of ICI in first-line for MSI/dMMR mCRC patients. The 3-arm COMMIT trial was initially a three-arm study (mFOLFOX6 plus bevacizumab versus mFOLFOX6 plus bevacizumab and atezolizumab versus atezolizumab alone). It was redesigned in June 2020 following the presentation of the KEYNOTE-177 results: the mFOLFOX6 plus bevacizumab arm was closed. The 3-arm CA209-8HW trial, in a 1/2/2 randomization schedule, compares standard-of-care first-line chemotherapy plus targeted therapy versus nivolumab alone versus nivolumab plus ipilimumab.

## 3. Patients with ICI-Resistant MSI/dMMR mCRC

Despite high rates of response and durable clinical benefit with ICIs, around 40 to 50% of MSI/dMMR mCRC exhibit primary resistance to anti-PD1 or anti-PDL1 monotherapy. First, pseudoprogression occurs in MSI/dMMR mCRC patients treated with ICIs in 10% in our experience and is more frequent with anti-PD1 compare to combination of PD1 and anti CTL4. Pseudoprogression in our experiences is an early phenomena that represents most of primary radiological progressions and needs to be considered in clinical practice to avoid prematurely discontinuation of ICIs [27].

Second, a significant amount of these refractory tumors is mistakenly diagnosed as MSI/dMMR [28,29]. It is therefore mandatory (i) to properly analyze the MSI/dMMR status with gold-standard methods (immunohistochemistry, pentaplex PCR) and (ii) to properly analyze the reports of these tests to detect any potential misinterpretation and discrepancy.

Preliminary results on potential biomarkers for the efficacy of ICIs amongst MSI/dMMR mCRC did not found significant predictive impact of *RAS*/*RAF* mutational status, the inherited (i.e., Lynch syndrome) or sporadic origin of MMR deficiency [13,15,17,22]. To note, the analyses of ICI efficacy among Lynch-related cancers and sporadic cases are probably imperfect, since the characterization of Lynch syndrome as positive or negative is generally determined by investigators based on pas medical history collected clinical records without mandatory genetic testing, which is known to be inaccurate [1,17,32]. Surprisingly, the analysis of the *KRAS* mutated subgroup in the KEYNOTE-177 study suggested a reduced activity of pembrolizumab compared to chemotherapy in this population. Nonetheless, the objective response rate with nivolumab plus ipilimumab as first-line treatment in the CheckMate-142 study was consistent across mutational subgroups [33]. Tumor infiltrating lymphocytes has been suggesting as an interesting parameter in a small cohort study [28]. The impact of tumor mutational load remains controversial within the MSI/dMMR population: positive correlations between tumor mutational load and the efficacy of ICI were observed but the sample sizes remain small, with potential tumors misdiagnosed as MSI/dMMR amongst ICI-resistant cases [34,35]. *Beta-2-microglobulin* mutations are not associated with lack of efficacy of ICI for MSI/dMMR mCRC patients [36]. Yet, MSI/dMMR tumors frequently display truncating *beta-2-microglobulin* mutations that have been implicated as causes of acquired resistance to immunotherapy in melanoma [37,38]. Finally, there is no evidence the CMS classification or the *BRAF*^V600E^-mutated molecular subtypes (i.e., BM1 and BM2) might help identifying ICI-resistant cases among the MSI/dMMR population [39,40]. Furthermore, the applicability of the CMS classification in metastatic setting is controversial due to spatial heterogeneity and CMS modifications under the pressure of anticancer treatments [41,42]. All in all, it is now necessary to develop screens at both the exome and transcriptome levels with the aim of identifying, without a priori, the resistance mechanisms to ICI in MSI/dMMR tumors. Such studies will be complementary to the ones in the literature, which have focused primarily on candidate gene approaches.

Combination of ICIs seems to decrease resistance to anti-PD(L)1 antibodies. The addition of ipilimumab to nivolumab seems to improve the clinical outcomes of ICI-naïve MSI/dMMR mCRC patients, with 12% of primary resistance compared to 31% with nivolumab alone [15], but phase III studies are waiting especially the CA209-8HW trial, comparing nivolumab to nivolumab and ipilimumab. Furthermore, some reports suggest that ICI combinations might be efficient for MSI/dMMR cancer patients resistant to anti-PD(L)1 [43,44]. Ipilimumab alone or in combination with nivolumab has been evaluated as a salvage therapy after anti-PD(L)-1 failure for patients with advanced melanoma. In a retrospective study (*n* = 19), it led to a 58% disease control rate with 11% of objective [45]. The SWOG S1616 phase II study is currently assessing the efficacy of ipilimumab ± nivolumab in patients with advanced melanoma refractory to a PD-1 inhibitor (NCT03033576). In another retrospective series of patients with metastatic renal cell carcinoma who received prior anti–PD-1 pathway-targeted therapy and subsequently received ipilimumab and nivolumab, the objective response rate was 20% [46]. TITAN RCC is a phase 2 study of nivolumab monotherapy with additional nivolumab/ipilimumab “boost” cycles in advanced renal cell carcinoma patients. In this trial, approximately 10% of patients who did not respond to nivolumab alone had a subsequent response, or were “rescued”, after receipt of nivolumab plus ipilimumab [47].

Data are lacking concerning the biology of MSI/dMMR tumors harboring ICI resistance. In other tumor locations such as melanoma, acquired resistance to ICI has been associated with increased signaling of the immunosuppressive TGF-β pathway [48,49,50,51]. Still, in a phase II study evaluating dual inhibition of TGF-β and PD-L1 by bintrafusp alfa for patients with MSI/dMMR metastatic solid tumors who had progressed on prior ICI, the response rate was 0%, and the disease control rate was 21% [52]. Anyhow, longitudinal monitoring of tumor cells and tumor microenvironment is a key point to gain knowledge about tumor resistance [53]. This is even more relevant since tumor microenvironment might explain the 8% rate of dissociated responses in cancer patients treated with ICI [54,55]. Notably, several reports suggest the adrenal glands might act as a sanctuary site for ICI-treated cancer cells. Our group recently reported a case series of 5 ICI-treated MSI/dMMR mCRC patients who experienced disease progression limited to the adrenal gland [56]. The molecular investigations performed for one patient suggest an impairment of the antigen-presentation pathway in relation the endogenous production of glucocorticoids, though the inhibition of the *NF-κB* gene, controlling the HLA-class I expression, or through adrenal-specific mutations in glucocorticoid-target genes involved in the antigen presentation pathway. These results deserved to be confirmed in larger cohorts. Nonetheless, the 8% rate of dissociated responses for ICI-treated cancer patients suggest these patients should be managed in a multidisciplinary approach. While the benefit-risk balance appears against the surgical management of post-ICI residual lesions given the high rate of complete pathological response in this clinical situation [57], it seems appropriate to combine ICI with local treatments for patients experiencing ICI-associated dissociated responses as for oligometastatic diseases [58].

## 4. Conclusions

ICIs have become the new standard of care for patients with MSI/dMMR mCRC, with labelling in the US for all lines but only in first-line in Europe. Many questions remain unresolved for the clinical management of these patients. Predictive biomarkers of resistance to ICI among MSI/dMMR tumors are urgently needed in order to (i) choose the best treatment for these patients (anti-PD(L)1 monotherapy or combined with anti-CTLA4 or chemotherapy ± anti-PD(L)1), and (ii) develop new therapeutic strategies for MSI/dMMR mCRC patients who had progressed on prior ICI. The development of ICI in adjuvant and neoadjuvant settings is also of great interest in the MSI/dMMR population, even more since a significant proportion of these patients are Lynch syndrome carriers, at high risk of developing cancers in their lifetime.

## Figures and Tables

**Table 1:** Immune checkpoint inhibitors in MSI/dMMR metastatic colorectal cancer.

Clinical Trials	Prior Systemic Treatment	*n*	CR (%)	PR (%)	SD (%)	PD (%)	NE (%)	1-Year PFS Rate (%)	1-Year OS Rate (%)	2-Year PFS Rate (%)	2-Year OS Rate (%)	Median Follow-Up (Months)
Keynote-016 [11]												
Pembrolizumab	≥1	28	11	46	32	4	7	-	-	-	-	8.7
Keynote-164 [12,13,14]												
Pembrolizumab, cohort A	≥2	61	3	30	18	46	3	34	72	31	55	31
Pembrolizumab, cohort B	≥1	63	8	25	24	40	3	41	76	37	63	24
CheckMate-142 [15,16,17,18]												
Nivolumab	≥1	74	9	24	31	31	5	44	-	-	-	21
Nivolumab + Ipilimumab	≥1	119	6	52	28	12	3	71	85	60	74	25.4
Nivolumab + Ipilimumab	0	45	13	56	16	13	2	77	83	74	79	29.0
NIPICOL [19]												
Nivolumab + Ipilimumab	≥2	57	19	40	30	5	3	73	84	-	-	18.1
CD-ON-MEDI4736-1108 [20]												
Durvalumab	≥1	36	22		-	-	-	38			54	29
NCT02227667 [20]												
Durvalumab	≥1	11	27		-	-	-	36				30
GARNET [21]												
Dostarlimab	≥1	69	36		-	-	-	-	-	-	-	-
